# Supplementary material for: Hybrid Immunity Results in Enhanced and More Sustained Antibody Responses after the Second Sinovac-CoronaVac Dose in a Brazilian Cohort: DETECTCoV-19 Cohort
Source: Viruses. 2023 Sep 23;15(10):1987. doi: 10.3390/v15101987 (PMC10610994; doi:10.3390/v15101987)
Supplement: Supplementary file 1 [file viruses-15-01987-s001.zip › viruses-2581876-supplementary.pdf]

Supplementary Table S1. Characteristics and Serological testing comparing Naïve and COVID-19 previously exposed individuals.

		COVID-19 (n=22)	Naïve (n=48)	P value
**Characteristics**				
Female, n (%)		17 (77.3)	19 (39.6)	0.0034*
Age, mean		45.3	39.0	0.0688‡
Age, median (IQR)		45.5 (28.75 – 63)	37 (30 – 52)	
Age, range		23 – 69	22 – 57	
Income	0 – 3 minimum salaries	6 (27.3)	7 (14.6)	0.0082*
	4 – 6 minimum salaries	11 (50.0)	11 (22.9)	
	> 6 minimum salaries	5 (22.7)	30 (62.5)	
Comorbidities, yes, n (%)		15 (68.2)	25 (52.1)	0.2064*
	Diabetes	4 (18.2)	4 (8.3)	
	Hypertension	8 (36.4)	12 (25.0)	
	Obesity	3 (13.6)	10 (20.8)	
	Asthma	4 (18.2)	3 (6.25)	
	Cardiopathy or nephropathy	1 (4.5)	2 (4.2)	
**Serological testing**				
Before vaccination	Anti-Nucleocapsid IgG positive, n (%)	20 (90.9)	1 (2.1)	<0.0001‡
	Anti-Nucleocapsid IgG (RI), median (IQR)	5.24 (3.4–8.05)	0.92 (0.62–1.17)	<0.0001‡
	Anti-Spike-RBD IgG positive, n (%)	18 (81.8)	2 (4.2)	<0.0001‡
	Anti-Spike-RBD IgG (BAU/mL), median (IQR)	108.1 (66.2–404.4)	10.34 (8.9– 12.4)	<0.0001‡
	RBD-ACE2 inhibition (%), median (95% IC)	24.56 (15.6–49.3)	-0.54 (-3.2–1.5)	<0.0001‡
Peak response after fully vaccinated$	Anti-Nucleocapsid IgG positive, n (%)	22 (100.0)	33 (68.75)	0.0075‡
	Anti-Nucleocapsid IgG (RI), median (IQR)	8.93 (7.3–11.33)	3.04 (1.3–6.4)	<0.0001‡
	Anti-Spike-RBD IgG positive, n (%)	22 (100.0)	44 (91.7)	0.4811‡
	Anti-Spike-RBD IgG (BAU/mL), median (IQR)	434.8 (222.8–735.6)	172.0 (79.5–343.9)	0.0104‡
	RBD-ACE2 inhibition (%), median (95% IC)	83.73 (64.4–95.7)	34.65 (17.05–61.5)	<0.0001‡

$15 days after second dose of vaccine was considered fully vaccinated. RI: Reactivity Index. BAU: binding antibodies units. IQR: interquartile. IgG: immunoglobulin G. RBD: receptor binding domain. ACE2: angiotensin-converting enzyme-2. ‡ Unpaired T test. *Chi-square test.

Supplementary Table S2. Characteristics and Serological testing comparing CoronaVac and AstraZeneca-vaccinated individuals.

		CoronaVac (n=67)	AstraZeneca (n=67)	P value
**Characteristics**				
Female, n (%)		34 (50.75)	41 (61.2)	0.2232*
Age, mean		40.78	45.57	0.0174‡
Age, median (IQR)		37.0 (30.0 – 54.0)	46.0 (38.0 – 53.0)	
Age, range		22 – 69	25 – 69	
Income	0 – 3 minimum salaries	12 (17.9)	7 (10.4)	0.3029*
	4 – 6 minimum salaries	21 (31.3)	18 (26.9)	
	> 6 minimum salaries	34 (50.8)	42 (62.7)	
Commorbities, yes, n (%)		38 (56.7)	42 (62.7)	0.4811*
Diabetes		8 (11.9)	2 (3.0)	
Hypertension		19 (28.4)	7 (10.4)	
Obesity		12 (17.9)	6 (9.0)	
Asthma		5 (7.5)	6 (9.0)	
Cardiopath or nephropath		2 (3.0)	1 (1.5)	
Hypothyroidism		1 (1.5)	1 (1.5)	
Neoplasms or Immunosuppressions		2 (3.0)	4 (6.0)	
COVID-19 prior to vaccination, yes, n (%)		20 (29.9)	33 (49.3)	0.0216*
**Serological testing**				
After fully vaccinated$	Anti-Spike-RBD IgG positive, n (%)	63 (94.0)	67 (100.0)	0.0423‡
	Anti-Spike-RBD IgG (BAU/mL), median (IQR)	175.9 (110.5–420.1)	621.9 (318.5–1115)	0.0013‡
	RBD-ACE2 inhibition (%), median (95% IC)	47.12 (34.5–60.1)	88.92 (77.8–93.7)	<0.0001‡

$15 days after second dose of vaccine was considered fully vaccinated. BAU: binding antibodies units. IQR: interquartile. IgG: immunoglobulin G. RBD: receptor binding domain. ACE2: angiotensin-converting enzyme-2. ‡ Unpaired T test. *Chi-square test.

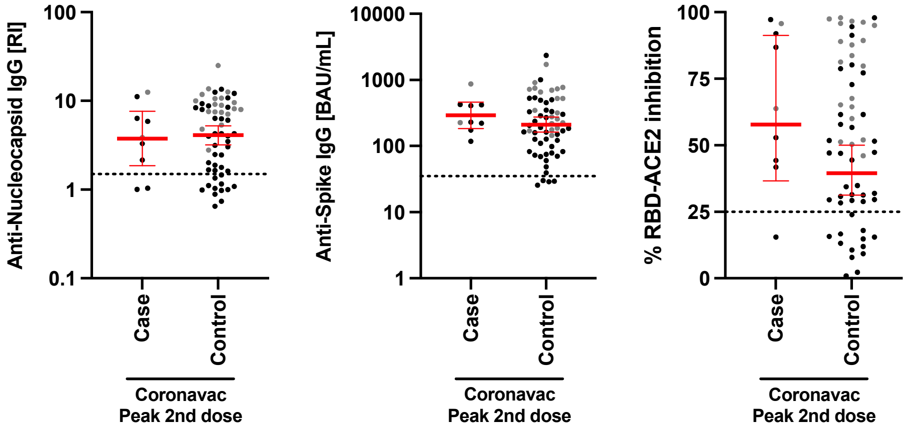

Supplementary Figure S1: Equivalent humoral response between Sinovac-CoronaVac vaccine breakthrough cases and controls.
Symptomatic vaccine breakthrough cases (n=9) were paired with age, sex, time of vaccination and previous COVID-19 history to vaccinated controls (n=61) without breakthrough infection during the same period. Immunoassays measured serum anti-nucleocapsid IgG, anti-Spike-RBD IgG and %RBD-ACE2 inhibitory antibodies before and after immunization. Peak humoral response after two doses. Median is represented by red horizontal lines. Horizontal dotted lines represent assay cut-off. Each patient represents one data point in the graph.

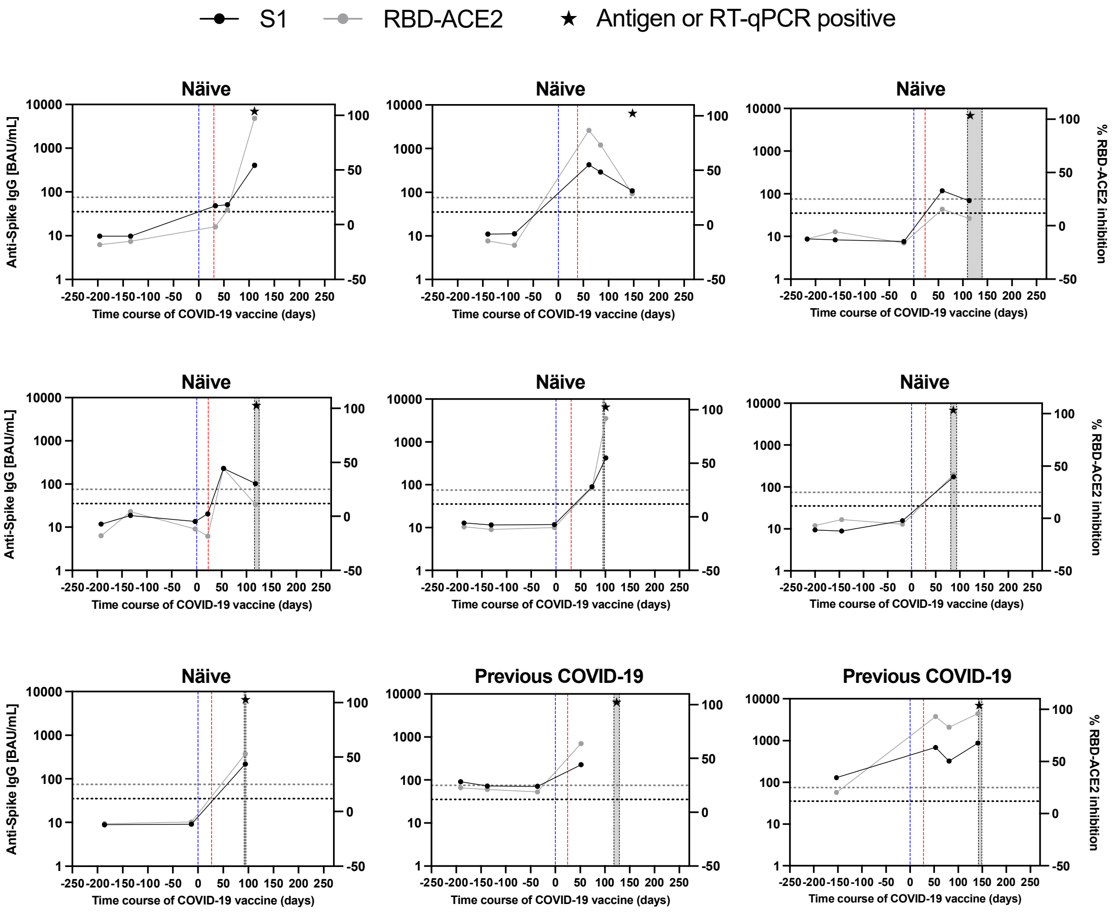

Supplementary Figure S2: Breakthrough cases

Each graph represents one symptomatic patient diagnosed after two doses of Sinovac-CoronaVac vaccine. Left and right X-axis depict anti-Spike-RBD IgG and %RBD-ACE2 inhibitory antibodies, respectively. Grey-line and black-line in graphs represents anti-Spike-RBD IgG and %RBD-ACE2 inhibitory antibodies, respectively. Blue vertical line represents first dose and red-line second vaccine dose. Asterisk depicts day tested positive by RT-PCR for SARS-CoV-2. Grey-area depicts days with symptoms.

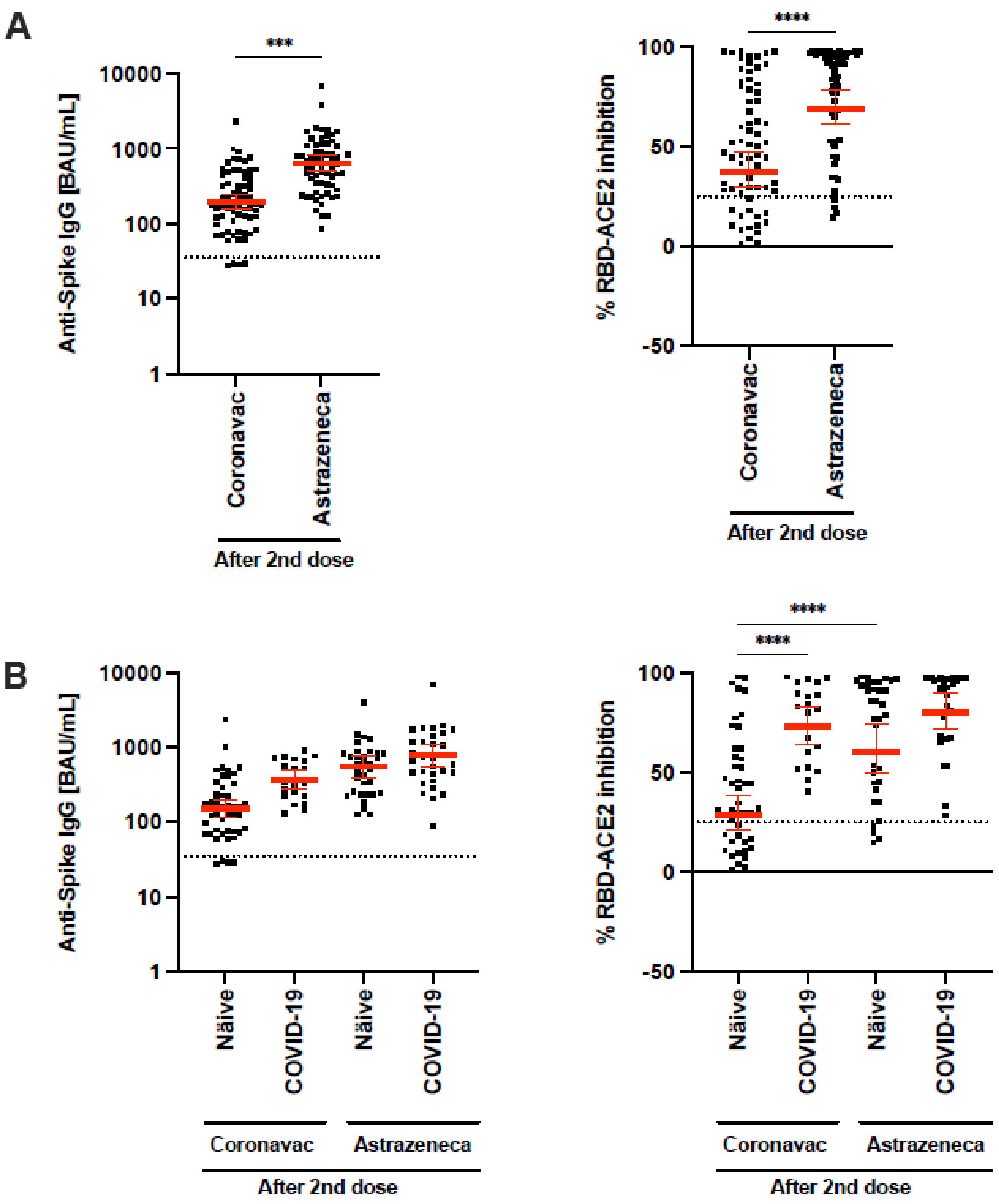

Supplementary Figure S3: Adenovirus-vectored SARS-CoV-2 vaccine response was superior to inactivated SARS-CoV-2 vaccine response. Immunoassays measured anti-Spike-RBD IgG and %RBD-ACE2 inhibitory antibodies before and after immunization. (A) Peak humoral response after two doses among Sinovac-CoronaVac (n=61) and AstraZeneca (n=66) vaccine was compared. (B) Vaccinees were stratified as naïve and infected individuals to compare humoral response compared. Median is represented by red horizontal lines. Horizontal dotted lines represent assay cut-off. Each patient represents one data point in the graph. One-way ANOVA test with Tukey's post hoc test was used. *** $p < 0.001$, **** $p < 0.0001$.
